# Changes in Drug Utilization during a Gap in Insurance Coverage: An Examination of the Medicare Part D Coverage Gap

**DOI:** 10.1371/journal.pmed.1001075

**Published:** 2011-08-16

**Authors:** Jennifer M. Polinski, William H. Shrank, Haiden A. Huskamp, Robert J. Glynn, Joshua N. Liberman, Sebastian Schneeweiss

**Affiliations:** 1 Division of Pharmacoepidemiology and Pharmacoeconomics, Brigham and Women's Hospital, Boston, Massachusetts, United States of America; 2 Harvard Medical School, Boston, Massachusetts, United States of America; 3 Division of Preventive Medicine, Brigham and Women's Hospital, Boston, Massachusetts, United States of America; 4 Department of Biostatistics, Harvard School of Public Health, Boston, Massachusetts, United States of America; 5 CVS Caremark, Hunt Valley, Maryland, United States of America; Harvard School of Public Health, United States of America

## Abstract

**Background:**

Nations are struggling to expand access to essential medications while curbing rising health and drug spending. While the US government's Medicare Part D drug insurance benefit expanded elderly citizens' access to drugs, it also includes a controversial period called the “coverage gap” during which beneficiaries are fully responsible for drug costs. We examined the impact of entering the coverage gap on drug discontinuation, switching to another drug for the same indication, and drug adherence. While increased discontinuation of and adherence to essential medications is a regrettable response, increased switching to less expensive but therapeutically interchangeable medications is a positive response to minimize costs.

**Methods and Findings:**

We followed 663,850 Medicare beneficiaries enrolled in Part D or retiree drug plans with prescription and health claims in 2006 and/or 2007 to determine who reached the gap spending threshold, *n = *217,131 (33%). In multivariate Cox proportional hazards models, we compared drug discontinuation and switching rates in selected drug classes after reaching the threshold between all 1,993 who had no financial assistance during the coverage gap (exposed) versus 9,965 multivariate propensity score-matched comparators with financial assistance (unexposed). Multivariate logistic regressions compared drug adherence (≤80% versus >80% of days covered). Beneficiaries reached the gap spending threshold on average 222 d ±79. At the drug level, exposed beneficiaries were twice as likely to discontinue (hazard ratio [HR]  = 2.00, 95% confidence interval [CI] 1.64–2.43) but less likely to switch a drug (HR  = 0.60, 0.46–0.78) after reaching the threshold. Gap-exposed beneficiaries were slightly more likely to have reduced adherence (OR  = 1.07, 0.98–1.18).

**Conclusions:**

A lack of financial assistance after reaching the gap spending threshold was associated with a doubling in discontinuing essential medications but not switching drugs in 2006 and 2007. Blunt cost-containment features such as the coverage gap have an adverse impact on drug utilization that may conceivably affect health outcomes.

*Please see later in the article for the Editors' Summary*

## Abstract 2

Jennifer Polinski and colleagues estimated the effect of the "coverage gap" during which US Medicare beneficiaries are fully responsible for drug costs and found that the gap was associated with a doubling in discontinuing essential medications.

## Editors' Summary

**Background:**

Every year, more effective drugs for more diseases become available. But the availability of so many drugs poses a problem. How can governments provide their citizens with access to essential medications but control drug costs? Many different approaches have been tried, among them the “coverage gap” or “donut hole” approach that the US government has incorporated into its Medicare program. Medicare is the US government's health insurance program for people aged 65 or older and for younger people with specific conditions. Nearly 50 million US citizens are enrolled in Medicare. In 2006, the government introduced a prescription drug insurance benefit called Medicare Part D to help patients pay for their drugs. Until recently, beneficiaries of this scheme had to pay all their drug costs after their drug spending reached an initial threshold in any calendar year ($2,830 in 2010). Beneficiaries remained in this coverage gap (although people on low incomes received subsidies to help them pay for their drugs) until their out-of-pocket spending reached a catastrophic coverage spending threshold ($4,550 in 2010) or a new year started, after which the Part D benefit paid for most drug costs. Importantly, the 2010 US health reforms have mandated a gradual reduction in the amount that Medicare Part D enrollees have to pay for their prescriptions when they reach the coverage gap.

**Why Was This Study Done?:**

Three to four million Medicare Part D beneficiaries reach the coverage gap every year (nearly 15% of all Part D beneficiaries). Supporters of the coverage gap concept argue that withdrawal of benefits increases beneficiaries' awareness of medication costs and encourages switching to cost-effective therapeutic options. However, critics argue that the coverage gap is likely to lead to decreased drug utilization, increased use of health services, and adverse outcomes. In this study, the researchers examine the impact of entering the coverage gap on drug discontinuation, switching to another drug for the same indication, and drug adherence (whether patients take their prescribed drugs regularly).

**What Did the Researchers Do and Find?:**

The researchers studied 663,850 Medicare beneficiaries enrolled in Part D or in retiree drug plans (which provide coverage under a employer's group health plan after retirement; the retiree drug plans included in this study did not have coverage gaps) who made prescription claims in 2006 and/or 2007. A third of these individuals reached the gap spending threshold. The researchers used detailed statistical analyses to compare the drug discontinuation, switching, and adherence rates of 1,993 beneficiaries who had no financial assistance during the coverage gap (exposed beneficiaries) with those of 9,965 matched beneficiaries who had financial assistance during the coverage gap (unexposed). On average, beneficiaries reached the gap spending threshold 222 days into the year (mid August). In a drug-level analysis, exposed beneficiaries were twice as likely to discontinue a drug and slightly more likely to have reduced drug adherence than unexposed beneficiaries but 40% less likely to switch a drug after reaching the threshold. Similar results were obtained in a beneficiary-level analysis in which discontinuation, switching, and adherence rates were considered in terms of the complete drug regimen of individual beneficiaries.

**What Do These Findings Mean?:**

These findings show that, among the Medicare beneficiaries investigated, a lack of financial assistance to pay for drugs after reaching the coverage gap spending threshold led to a doubling in the rate of drug discontinuation and a slight reduction in drug adherence. Surprisingly, lack of financial assistance resulted in a decrease in drug switching even though the Centers for Medicare and Medicaid Services advise patients to consider switching to generic or low-cost drugs. Importantly, the researchers estimate that, for the whole Medicare population, the lack of financial assistance to pay for drugs could result in an additional 18,000 patients discontinuing one or more prescription drug per year. Although this study did not directly investigate the effect of the coverage gap on patient outcomes, these findings suggest that this and other blunt cost-containment approaches could adversely affect health outcomes through their effects on drug utilization. Thus, insurance strategies that specifically promote the use of drugs with high benefit but low cost might be a better approach for governments seeking to improve the health of their citizens while reining in drug costs.

**Additional Information:**

Please access these websites via the online version of this summary at http://dx.doi.org/10.1371/journal.pmed.1001075.

- The US Department of Health and Human Services Centers for Medicare and Medicaid provides information on all aspects of Medicare, including general advice on bridging the coverage gap and an information sheet on bridging the coverage gap in 2011
- Medicare.gov, the official US government website for Medicare, provides information on all aspects of Medicare (in English and Spanish), including a description of Part D prescription drug coverage
- An information sheet from the Kaiser Family Foundation explains the key changes to the Medicare Part D drug benefit coverage gap that were introduced in the 2010 health care reforms
- MedlinePlus provides links to further information about Medicare (in English and Spanish)

## Introduction

Internationally, governments are wrestling with the seemingly contradictory goals of expanding citizens' access to essential medications while at the same time controlling rising drug costs. Multiple benefit designs have been proposed and implemented by diverse countries such as Canada, China, Australia, Germany, and the United Kingdom, including reference pricing [1],[2], generic substitution [3], income-based deductibles [4], copayments and coinsurance [5],[6], incentive-based tiered formularies [7], negative and positive subsidy lists [8], prescribing budgets [9],[10], and drug caps [11], each with varied success. In 2006, the US government expanded its Medicare health insurance program for the elderly to offer a prescription drug insurance benefit, Medicare Part D. Part D's goal was to improve beneficiaries' access to and the affordability of essential medications, but the US government was also mindful of the program's budgetary impact. Therefore, the standard Part D benefit design included a novel cost containment feature, the “coverage gap.”

After drug spending reaches an initial threshold ($2,830 in 2010) in a calendar year, beneficiaries enter the coverage gap, a period during which they are responsible for 100% of drug costs. Beneficiaries remain in the coverage gap period until out-of-pocket drug spending reaches a catastrophic coverage spending threshold ($4,550 in 2010) at which time cost-sharing is dramatically reduced, or until the benefit resets at the next calendar year [12]. Of note, low-income beneficiaries receive subsidies to help them pay for drugs and thus are not 100% responsible for their drug costs during the coverage gap period.

Between 2.9–3.8 million (11%–14%) Medicare Part D beneficiaries reach the coverage gap each year and receive no financial assistance to help pay for drugs during this period [13],[14]. Proponents have argued that the coverage gap could help both beneficiaries and the US health care system save money by increasing beneficiaries' awareness of medication costs and encouraging switching to or new selection of cost-effective therapeutic options [15]. Critics point to evidence that similar drug caps and increases in cost-sharing have been associated with decreased drug utilization, increased health services use, and adverse outcomes [6],[11],[16]–[18].

To date, researchers have observed reduced drug utilization and adherence among beneficiaries enrolled in Medicare Advantage plans who reach the coverage gap spending threshold and had no financial assistance to pay for drugs [19]–[22]. Zhang et al. noted a 14% reduction in drug utilization among beneficiaries with no financial assistance during the coverage gap [22]. These beneficiaries also reduced their adherence to chronic medications 3%–8% during the coverage gap compared to the precoverage gap period [20] and were 17% less likely than beneficiaries who had financial assistance to be adherent to their medications during the coverage gap period [19]. While important contributions to the field, results from these Medicare Advantage-based studies may not be generalizable to the 70% of all Part D beneficiaries enrolled in stand-alone Part D plans [23]. Unlike stand-alone plans that only provide drug coverage, Medicare Advantage plans manage health and drug insurance benefits, and so may have different incentives in terms of coverage and benefit design. The remaining study of beneficiaries' coverage gap behavior found that among those who reached the coverage gap, 20% discontinued, switched, or reduced their medication use [13]. However, this study did not employ a comparator group nor link prescription data to clinical information, both important steps in establishing baseline rates of utilization and minimizing confounding.

In this study, we used nationally representative cohorts of Medicare beneficiaries who enrolled in one of 182 stand-alone Part D plans or in retiree plans with drug coverage. We assessed the characteristics of beneficiaries who reached the coverage gap spending threshold and determined their time to reach the threshold. Among those who reached the threshold, we compared rates of drug discontinuation and switching and the odds of reduced drug adherence between those who were 100% responsible for their drug costs during the coverage gap and those who received financial assistance to pay for drugs during this time. We hypothesized that compared to beneficiaries who received financial assistance, beneficiaries who were fully responsible for their drug costs during the coverage gap would be more likely to discontinue medications but less likely to switch from one medication to a second, potentially less costly medication with the same indication for use. We also hypothesized that beneficiaries would be less adherent to their medications if they had no financial assistance during the gap. Our study aimed to provide information about the coverage gap's influence on beneficiaries' drug utilization behaviors and to evaluate the applicability of the coverage gap design to other insurance settings.

## Methods

### Ethics Statement

The Human Subjects Committee at Brigham and Women's Hospital approved this study. Because the study was a secondary analysis of previously collected data, both written and oral consent requirements were waived. Data use agreements were in place with all data providers.

### Data Sources and Study Population

We studied community-dwelling, fee-for-service Medicare beneficiaries with prescription drug coverage through either a stand-alone Part D plan or a retiree drug plan in 2006 or 2007 that was administered by CVS Caremark, a pharmacy benefits management company that adjudicates approximately 660 million prescriptions per year [24]. Medicare beneficiaries' Caremark prescription drug claims were linked to Medicare Parts A, B, and enrollment data to obtain diagnostic, health care utilization, and demographic information. Part D plans were characterized as providing no or generic-only drug coverage during the coverage gap. None of the retiree plans had a coverage gap feature.

We established two cohorts of beneficiaries age 65 or older. Because Part D did not begin until 2006, beneficiaries in the “Early Part D*”* cohort (2005–2006) had no Caremark drug claims for 2005, the baseline year, but had claims in the study year, 2006. Therefore, we required ≥1 prescription claim in 2006. Beneficiaries in the “Established Part D*”* cohort (2006–2007) had continuous Caremark eligibility and ≥1 prescription drug claim in both the baseline, 2006, and the study year, 2007. Both cohorts had Medicare eligibility and ≥1 inpatient or outpatient health care claim in both the baseline and study years.

We used plan enrollment and beneficiaries' out-of-pocket drug spending in the study year to categorize beneficiaries into four groups. Of the three Part D groups, two received subsidies to defray cost-sharing. Full subsidy beneficiaries had incomes ≤$7,500 in 2006 or ≤$7,620 in 2007 and per prescription cost-sharing that did not exceed $5 in 2006 or $5.35 in 2007, even when in the coverage gap. Partial subsidy beneficiaries had higher incomes ($7,501–$11,500 in 2006, $7,620–$11,710 in 2007) and cost-sharing ≤15% for each prescription in both the initial coverage and coverage gap periods. In contrast, the third Part D group, nonsubsidy enrollees, exceeded these income limits and was responsible for 100% of drug costs in the coverage gap. Retirees enrolled in retiree plans, none of which had a coverage gap design or benefit cap, comprised the final group and thus always had financial assistance to pay for drugs. Assignment algorithm details are in Text S1.

We hypothesized that a beneficiary's plan enrollment and subsequent drug utilization were good predictors of whether he would reach the coverage gap spending threshold; however, baseline year drug use was not available for the Early Part D cohort. To ensure comparable drug data from both cohorts, we limited our cohorts to beneficiaries who reached the threshold ≥60 d after plan enrollment.

In total, 663,850 beneficiaries met inclusion and exclusion criteria. Using beneficiaries' and plans' drug spending in study years 2006 and 2007, we further limited our primary study cohort to the 217,131 (33%) beneficiaries who reached the coverage gap spending threshold in each year (cumulative spending of $2,250 in 2006; $2,400 in 2007).

### Study Design and Exposures

**Figure 1:**
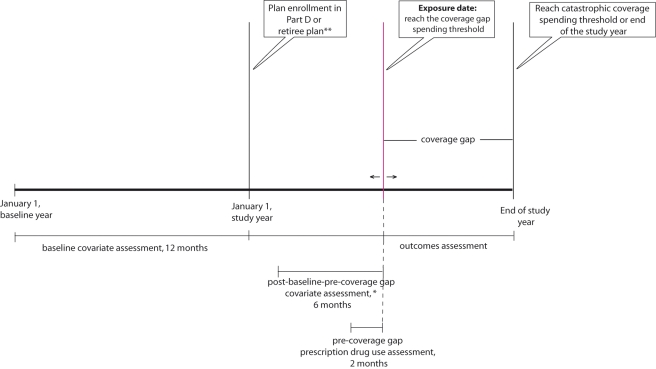
Prospective open cohort study design.

To assess drug utilization changes after reaching the coverage gap spending threshold, we conducted two prospective open cohort studies (Figure 1 **).** In both cohorts, baseline covariates were assessed in the 12 mo prior to plan enrollment. We classified beneficiaries as “exposed” if they received no financial assistance to pay for drug costs in the coverage gap (i.e., the nonsubsidy enrollees), and “unexposed” otherwise (full subsidy, partial subsidy, and retirees). If a nonsubsidy enrollee was in a Part D plan with generic drug coverage during the coverage gap but was responsible for 100% of branded drug costs, he was also classified as exposed. In sensitivity analyses, these 12 beneficiaries with generic drug coverage were removed. All beneficiaries entered the study on the date when they reached the coverage gap spending threshold and were censored on the date of a study outcome of interest, death, nursing home admission, hospitalization >14 d, reaching the catastrophic coverage spending threshold, or on December 31 of the study year.

### Covariate Assessment and Propensity Score Matching

We used two steps to balance measured covariate distributions in the exposed and unexposed groups. First, we constructed a propensity score (PS) that assessed each beneficiary's propensity to receive financial assistance to pay for drug costs upon reaching the coverage gap spending threshold. PS models included age, gender, race, region of the US, rural/urban residence, median household income, Charlson comorbidity score [25], number of office-based drug infusions, physician visits and hospitalizations, Medicare inpatient and outpatient spending, diagnosis of cancer, rheumatoid arthritis (RA), cardiovascular conditions (atrial or ventricular fibrillation, hypertension, hyperlipidemia, hypercholesterolemia, myocardial infarction, angina, atherosclerosis, or congestive heart failure), depression, dementia, and/or diabetes, all assessed in the baseline year before a beneficiary enrolled in a Part D or retiree plan. Each exposed beneficiary was PS matched to five unexposed beneficiaries using a greedy matching algorithm [26]. PS model results are in Text S1.

Second, we measured additional covariates in the postbaseline-precoverage gap period. Beneficiaries' utilization during this period was likely a function of their health status, their drug plan's features, and their intuition as to whether they would reach the coverage gap spending threshold. In the 6 mo prior to reaching the threshold, we assessed the number of physician visits and hospitalizations, the Charlson comorbidity score, and days to reach the coverage gap spending threshold. In the 2 mo prior, we determined the number of unique drugs used and total drug spending. Follow-up for the adjusted analyses also began after beneficiaries reached the coverage gap spending threshold.

### Outcomes

We considered three outcomes, drug discontinuation, switching, and adherence, after a beneficiary reached the coverage gap spending threshold. We included any drug with available days' supply on the exposure date (date beneficiary reached the coverage gap spending threshold) for analysis, and only the first outcome observed on or after cohort entry was considered. In a first set of analyses, the unit of analysis was at the drug level. Drug X was discontinued if >30 d elapsed during the coverage gap when no drug X was available and no further fills of drug X were made during the coverage gap period. Drug X was switched if a beneficiary entered the coverage gap and switched from the generic to the brand version of drug X or vice versa, or stopped filling prescriptions for drug X but filled a new prescription for a drug with the same indication as drug X within 30 d after the days' supply of drug X was exhausted. Text S1 details acceptable switches. Drug adherence was measured using the proportion of days covered (PDC) [27], the number of drug X days supply available from cohort entry until censoring divided by the number of days from cohort entry until censoring. A beneficiary was considered adherent to drug X if PDC>80% and nonadherent otherwise.

For a second set of analyses at the beneficiary level, where a beneficiary might be taking one or more drugs, a beneficiary discontinued drugs if at least one of his available drugs was discontinued as described above. The beneficiary's date of discontinuation was the first date after reaching the coverage gap spending threshold on which there was no days' supply of the discontinued drug +30-d grace period. A beneficiary switched drugs if at least one of his available drugs was switched according to the definition above, with the switching date defined as the date of the first switch after cohort entry. Drug adherence was defined as a PDC ≥80% for all drugs a beneficiary was taking [28]. In sensitivity analyses at the drug and beneficiary level, we considered discontinuations and switches within 15 d and 45 d.

We focused on drugs used to treat one of five diseases of interest: RA, cardiovascular conditions, diabetes, depression, or dementia, each described in Text S1 **.** Self-injected drugs to treat RA are covered under Part D and their expense may move beneficiaries quickly into the coverage gap, while parenteral drugs are covered by the government [12]. Beneficiaries in the coverage gap may switch to parenteral drugs. Many drugs used to treat cardiovascular conditions, diabetes, and depression are available as lower-cost generics, so using generics may delay coverage gap entry, while switching to generics after coverage gap entry may reduce costs and minimize discontinuation. Finally, drugs to treat dementia are typically branded and expensive and questions persist as to their efficacy [29],[30].

### Statistical Analysis

Among our primary cohort who reached the coverage gap spending threshold, we cross-tabulated beneficiaries' characteristics at baseline by benefit group (full, partial, and nonsubsidy enrollees, retirees) and exposure status. We calculated the average time to reach the threshold among all beneficiaries and by beneficiary group, the proportion of beneficiaries who reached the threshold each month, and their top ten diagnoses.

Among exposed beneficiaries and multivariate PS-matched unexposed beneficiaries and with additional adjustment for postbaseline-precoverage gap covariates, we modeled the hazards of drug discontinuation and drug switching for each drug (drug-level analyses) using Cox proportional hazards models [31] and the odds of reduced drug adherence using logistic regression. As a sensitivity analysis to investigate concerns that the interdependence of the discontinuation and switching outcomes would result in overestimates of the hazards for each, we performed a competing risks analysis and calculated cumulative incidences and then the cumulative incidence ratio for each outcome, comparing the exposed with the unexposed [32],[33]. In beneficiary-level analyses, we ran stratified Cox proportional hazards models for the discontinuation and switching outcomes to allow for potentially different hazards among those taking various numbers of drugs. We used multivariate adjusted logistic regression to assess reduced drug adherence. In all beneficiary-level analyses, we employed robust standard errors to adjust for the correlation among multiple drugs used per person [34]. Subgroup analyses explored effect modification by drug class and generic/branded status, as measured by a Wald's test for the interaction. After testing for effect modification by cohort using a Wald's test for the interaction term, we also conducted pooled cohort analyses, estimating robust standard errors to account for correlation between beneficiaries present in both cohorts (drug-level and beneficiary-level analyses) as well as among multiple drugs used per person (beneficiary-level analyses) [34]. Finally, to estimate the population-level impact of exposure (having no financial assistance to help pay for drugs) during the coverage gap period, we calculated the covariate-adjusted rate differences for beneficiary-level drug discontinuation and switching between the exposed and unexposed in the pooled cohort using Poisson regression with robust standard errors. We then multiplied the rate differences for each outcome by the 11% prevalence of exposure and average 3.6-mo duration of the coverage gap as described by the Centers for Medicare and Medicaid Services [14]. The resulting estimates give the number and percentage of beneficiaries per year who would have a particular outcome in the total Medicare Part D beneficiary population due to exposure in the coverage gap period.

## Results

Among the 121,760 Early Part D and 95,371 Established Part D cohort beneficiaries who reached the coverage gap spending threshold, there were covariate imbalances across beneficiary groups, for example, female gender in the Early Part D cohort (76% of full subsidy versus 68% of partial subsidy, 64% of nonsubsidy enrollees, and 58% of retirees) and white race in 2006 (72% of full subsidy versus 93% of partial subsidy, 96% nonsubsidy enrollees, and 94% of retirees) (Table 1). There was a high prevalence of cardiovascular conditions (91%–95%) and diabetes (37%–56%) across groups. In the Established Part D cohort, the number of unique medications used in the baseline year varied from 5±1 among nonsubsidy enrollees to 9±4 among retirees.

**Table 1:** Baseline characteristics of 217,131 beneficiaries who reached the coverage gap spending threshold, by exposure status and benefit group.

Characteristics	Early Part D Cohort, 2005–2006, *n = *121,760				Established Part D Cohort, 2006–2007, *n = *95,371			
	Exposed	Unexposed			Exposed	Unexposed		
	Nonsubsidy	Full Subsidy	Partial Subsidy	Retirees	Nonsubsidy	Full Subsidy	Partial Subsidy	Retirees
*n (%) or mean* ± *SD unless otherwise noted*								
*n*	1,084	19,255	1,699	99,722	909	15,120	1,751	77,951
Female gender	689 (64)	14,634 (76)	1,153 (68)	56,754 (57)	603 (66)	11,464 (76)	1,153 (66)	43,959 (56)
Age (y) as of January 1, 2006	77±7	77±8	76±7	76±7	77±7	76±7	77±7	76±7
65–74	485 (45)	8,538 (44)	793 (47)	47,478 (48)	354 (39)	7,048 (47)	772 (44)	36,024 (46)
75–84	433 (40)	7,406 (38)	659 (39)	41,904 (42)	395 (43)	5,618 (37)	679 (39)	33,179 (43)
85+	166 (15)	3,311 (17)	247 (15)	10,340 (10)	160 (18)	2,454 (16)	300 (17)	8748 (11)
Race								
White	1,041 (96)	13,805 (72)	1,584 (93)	93,907 (94)	878 (97)	11,049 (73)	1,619 (92)	73,908 (95)
Black	30 (3)	3676 (19)	65 (4)	4,472 (4)	20 (2)	2,655 (18)	97 (6)	2,950 (4)
Other	13 (1)	1,774 (9)	50 (3)	1,343 (1)	11 (1)	1,416 (9)	35 (2)	1,093 (1)
Region								
Northeast	513 (47)	6,670 (35)	774 (46)	18,771 (19)	399 (44)	4,976 (33)	963 (55)	16,416 (21)
Central	210 (19)	5727 (30)	341 (20)	28,783 (29)	132 (15)	4,689 (31)	257 (15)	22,820 (29)
South	273 (25)	5,800 (30)	452 (27)	41,847 (42)	287 (32)	4,463 (30)	443 (25)	30,644 (39)
West	88 (8)	1,058 (5)	132 (8)	10,321 (10)	91 (10)	992 (7)	88 (5)	8,071 (10)
Urban residence	871 (80)	13,313 (69)	1,361 (80)	73,558 (74)	682 (75)	10,251 (68)	1,452 (83)	57,434 (74)
Median household income (US$)	50,708±	38,848±	48,724±	45,583±	49,558±	39,432±	51,759±	45,377±
	20,978	16,077	20,527	17,981	20,527	16,073	22,634	18,252
Total Medicare Parts A, B spending in the baseline year (US$)	4,606	5,844	6,000	3,452	4,465	5,704	6,882	3,565
(Median; IQR)	(1,959; 11,035)	(2,190; 15,040)	(2,479; 15,599)	(1,466; 9,286)	(2,012; 13,109)	(2,187; 14,654)	(2,877; 17,209)	(1521; 9,647)
Charlson comorbidity score	2±2	2±2	2±2	2±2	2±2	2±2	2±2	2±2
*n* physician visits	13±10	14±12	14±12	10±9	12±10	13±11	15±12	10±9
*n* hospitalizations	0.3±1	0.4±1	0.4±1	0.2±1	0.3±1	0.4±1	0.4±1	0.2±1
*n* office-based drug infusions	0.1±1	0.1±1	0.1±1	0.1±1	0.1±1	0.1±1	0.1±1	0.1±1
*n* unique drugs	—	—	—	—	5±1	6±3	6±3	9±4
Out-of-pocket drug spending (median; IQR)	—	—	—	—	794	51	946	561
					(445; 1,336)	(12; 81)	(285; 1,645)	(320; 973)
Plan drug spending (median; IQR)	—	—	—	—	902	2,604	1,094	3,055
					(635; 1,160)	(1,714; 3712)	(698; 1,865)	(2101; 4,429)
Diagnosis of cancer	223 (21)	2,470 (13)	303 (18)	17,929 (18)	175 (19)	1,946 (13)	342 (20)	14,153 (18)
Diagnosis of rheumatoid arthritis	42 (4)	814 (4)	80 (5)	3,419 (3)	40 (4)	671 (4)	104 (6)	2,960 (3)
Diagnosis of cardiovascular condition	1,014 (94)	18,089 (94)	1,589 (94)	90,452 (91)	844 (93)	14,230 (94)	1,665 (95)	70,892 (91)
Diagnosis of depression	123 (11)	5,204 (27)	337 (20)	10,193 (10)	98 (11)	3,861 (26)	321 (18)	8,279 (11)
Diagnosis of diabetes	436 (40)	10,729 (56)	832 (49)	36,394 (37)	349 (38)	8,501 (56)	859 (49)	29,736 (38)
Diagnosis of dementia	105 (10)	4,838 (25)	337 (20)	7,874 (8)	93 (10)	3,504 (23)	343 (20)	6,293 (8)

IQR, interquartile range; SD, standard deviation.

The top inpatient or outpatient diagnoses among beneficiaries who reached the coverage gap spending threshold in each 30-d period were remarkably consistent: anemia, chest pain, coronary atherosclerosis, uncontrolled diabetes, hypertension, hyperlipidemia, hypercholesterolemia, musculoskeletal pain, shortness of breath, and other malaise and fatigue (unpublished data).

**Figure 2:**
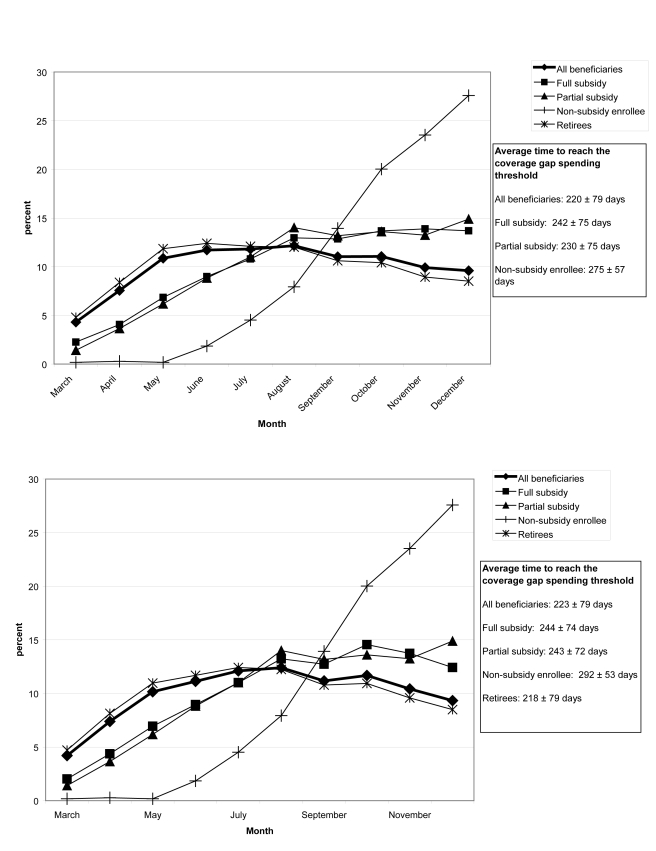
(A) Proportion of beneficiaries reaching the coverage gap spending threshold in each month in 2006, by beneficiary group. (B) Proportion of beneficiaries who reach the coverage gap spending threshold in each month in 2007, by beneficiary group.

In both 2006 and 2007, retirees reached the coverage gap spending threshold most quickly (Figures 2A and 2B), at an average of 215±80 d in 2006, whereas nonsubsidy enrollees took an average of 275±57 d in 2006. While the proportion of full subsidy, partial subsidy, and retirees' entering the coverage gap each month remained level over time, an increasing proportion of nonsubsidy enrollees entered over time, March  = 0.33%, September  = 12%, October  = 21% in 2007.

After PS matching, the measured covariate distributions were largely balanced between exposed and unexposed beneficiaries, with few residual differences (Table 2).

**Table 2:** Characteristics of multivariate propensity score-matched beneficiaries who reached the coverage gap spending threshold in the Early Part D cohort, 2006, or the Established Part D cohort, 2007.

Characteristics	Early Part D Cohort, 2006*n* = 6,504			Established Part D Cohort, 2007*n* = 5,454		
	Exposed (No Financial Assistance) *n* = 1,084	Unexposed (Receive Financial Assistance) *n* = 5,420	Delta	Exposed (No Financial Assistance) *n* = 909	Unexposed (Receive Financial Assistance) *n* = 4,545	Delta
*n (%) or mean ± SD*						
Female gender	689 (64)	3,439 (63)	−1%	603 (66)	2,996 (66)	0%
Age (y) as of January 1 of study year						
65–74	485 (45)	2,312 (43)	−2%	354 (39)	1,753 (39)	0%
75–84	433 (40)	2,253 (42)	+2%	395 (43)	1,984 (44)	+1%
85+	166 (15)	855 (16)	+1%	160 (18)	808 (18)	0%
Race						
White	1,041 (96)	5,198 (96)	0%	878 (97)	4,401 (97)	0%
Black	30 (3)	156 (3)	0%	20 (2)	100 (2)	0%
Other	13 (1)	66 (1)	0%	11 (1)	44 (1)	0%
Region						
Northeast	513 (47)	2,526 (47)	0%	399 (44)	1,998 (44)	0%
Midwest	210 (19)	1,085 (20)	+1%	132 (15)	723 (16)	+1%
South	273 (25)	1,415 (26)	+1%	287 (32)	1,373 (30)	−2%
West	88 (8)	394 (7)	−1%	91 (10)	451 (10)	0%
Charlson comorbidity score	2±2	2±2	0 points	2±2	2±2	0 points
*n* physician visits	13±10	13±12	0 visits	12±10	12±11	0 visits
*n* hospitalizations	0.3±1	0.3±1	0 hospitalizations	0.3±1	0.3±1	0 hospitalizations
*n* office-based drug infusions	0.1±1	0.1±1	0 infusions	0.1±1	0.1±1	0 infusions
Diagnosis of cancer	223 (21)	1,111 (21)	0%	175 (19)	838 (18)	−1%
Diagnosis of rheumatoid arthritis	42 (4)	205 (4)	0%	40 (4)	235 (5)	+1%
Diagnosis of cardiovascular condition	1,014 (94)	5,062 (93)	−1%	844 (93)	4,253 (94)	+1%
Diagnosis of depression	123 (11)	570 (11)	0%	98 (11)	468 (10)	−1%
Diagnosis of diabetes	436 (40)	2,181 (40)	0%	349 (38)	1,750 (39)	+1%
Diagnosis of dementia	105 (10)	527 (10)	0%	93 (10)	457 (10)	0%

SD, standard deviation.

In drug-level PS-matched analyses additionally adjusted for postbaseline–precoverage gap covariates, exposed beneficiaries were 2.00 (95% confidence interval [CI] 1.64–2.43) times more likely to discontinue a drug after reaching the coverage gap spending threshold than were unexposed beneficiaries (pooled cohort analyses, Table 3 **)**. There was a 2-fold increased hazard (hazard ratio [HR]  = 2.06, 1.68–2.53) of discontinuing cardiovascular drugs but no greater hazard of discontinuing oral hypoglycemic drugs (HR  = 1.86, 0.95–3.62). Exposed beneficiaries were 2.63 (1.93–3.58) times more likely to discontinue a branded cardiovascular drug but 1.79 (1.38–2.32) times more likely to discontinue a generic cardiovascular drug.

**Table 3:** Drug-level analyses.

Drug Changes	*HRs (95% CI)*					
	Early Part DCohort, 2006		Established Part D Cohort, 2007		Pooled Cohorts	
	Exposed (2,336)^a^	Unexposed (15,521)^a^	Exposed (1,841)^a^	Unexposed (13,037)^a^	Exposed (4,177)^a^	Unexposed (28,558)^a^
**Discontinue a drug**	**1.76 (1.34–2.32)**		**2.33 (1.76–3.08)**		**2.00 (1.64–2.43)**	
Discontinue a cardiovascular drug	1.94 (1.47–2.58)		2.20 (1.63–2.97)		2.06 (1.68–2.53)	
Discontinue a branded cardiovascular drug	1.81 (1.18–2.77)		4.48 (2.82–7.13)		2.63 (1.93–3.58)	
Discontinue a generic cardiovascular drug	2.02 (1.44–2.85)		1.51 (1.04–2.20)		1.79 (1.38–2.32)	
Discontinue an oral hypoglycemic drug	0.60 (0.17–2.08)		4.51 (1.97–10.35)		1.86 (0.95–3.62)	
**Switch a drug**	**0.75 (0.54–1.03)**		**0.52 (0.37–0.73)**		**0.60 (0.46–0.78)**	
Switch a cardiovascular drug	0.69 (0.47–1.02)		0.40 (0.23–0.70)		0.57 (0.41–0.79)	
Switch from a generic cardiovascular drug to a branded cardiovascular drug	0.90 (0.36–2.23)		0.38 (0.05–2.91)		0.72 (0.31–1.63)	
Switch from a branded cardiovascular drug to a generic cardiovascular drug	0.50 (0.23–1.08)		0.25 (0.06–1.05)		0.43 (0.22–0.84)	
Switch an oral hypoglycemic drug	1.08 (0.46–2.54)		0.32 (0.10–1.04)		0.59 (0.30–1.15)	
**Reduced adherence** b **to a drug**	**1.03 (0.91–1.17)**		**1.12 (0.97–1.30)**		**1.07 (0.98–1.18)**	
Reduced adherence to a cardiovascular drug	1.01 (0.87–1.17)		1.11 (0.94–1.30)		1.05 (0.94–1.17)	
Reduced adherence to an oral hypoglycemic drug	1.01 (0.68–1.50)		1.13 (0.72–1.78)		1.05 (0.78–1.42)	

Covariate-adjusted hazards of changes in drug discontinuation, switching, and covariate-adjusted odds of reduced drug adherence after reaching the coverage gap spending threshold among propensity score matched beneficiaries. Adjusted for the number of physician visits and hospitalizations, drugs used, drug spending, and Charlson comorbidity score in the postbaseline, predoughnut hole period after propensity score matching for baseline characteristics, which included: age, gender, race, region of the US, rural/urban residence, median household income, Charlson comorbidity score, number of office-based drug infusions, physician visits and hospitalizations, Medicare Parts A and B spending, and diagnosis of cancer, RA, cardiovascular conditions (atrial or ventricular fibrillation, hypertension, hyperlipidemia, hypercholesterolemia, myocardial infarction, angina, atherosclerosis, or congestive heart failure), depression, dementia, and/or diabetes.

a *n* Drugs available at cohort entry.

b Reduced adherence is defined as PDC <80%.

Although they discontinued drugs more often, exposed beneficiaries were less likely to switch a drug after reaching the coverage gap spending threshold than were unexposed beneficiaries, HR  = 0.60 (0.46–0.78). This decreased hazard of switching was consistent for cardiovascular drugs, HR  = 0.57 (0.41–0.79) but inconclusive for the oral hypoglycemic drugs, HR  = 0.59 (0.30–1.15). Exposed beneficiaries were 57% less likely to switch from a branded cardiovascular drug to a generic cardiovascular drug (0.22–0.84) than were unexposed beneficiaries. In the sensitivity analysis that accounted for the competing risk of drug discontinuation, exposed beneficiaries were also less likely to switch a drug after reaching the threshold than were unexposed beneficiaries, risk ratio  = 0.51 (unpublished data). Exposed beneficiaries showed increased odds of nonadherence to a drug after reaching the coverage gap as compared to unexposed beneficiaries, OR  = 1.07 (0.98–1.18), but these results were not significant. Sensitivity analyses with 15- and 45-d grace periods did not change discontinuation or switching results.

In beneficiary-level analyses (Table 4 **),** exposed beneficiaries had a 1.72 (1.36–2.16) times increased hazard of discontinuing at least one drug but a 40% (0.44–0.83) decreased hazard of switching at least one drug during the coverage gap period as compared to the unexposed, and 1.18 (1.05–1.32) times as likely to have decreased adherence for all their drugs as compared to the unexposed. When extrapolated to the larger population of all Medicare beneficiaries, entry into the coverage gap period with a lack of financial assistance to pay for drugs resulted in an additional 18,007 (9,432–33,442) beneficiaries (0.07%; 0.04%–0.13%) discontinuing at least one drug per year and 48,020 (40,302–54,880) fewer beneficiaries (0.18%; 0.15%–0.21%) switching at least one drug per year.

**Table 4:** Beneficiary-level analyses.

Drug Changes	Early Part DCohort, 2006		Established Part D Cohort, 2007		Pooled Cohorts	
	Exposed (897)^a^	Unexposed (4,769)^a^	Exposed (721)^a^	Unexposed (3,994)^a^	Exposed (1,618)^a^	Unexposed (8,763)^a^
*HRs (95% CI)*						
Discontinue ≥1 drug	1.63 (1.20–2.22)		1.79 (1.27–2.53)		1.72 (1.36–2.16)	
Switch ≥1 drug	0.74 (0.51–1.07)		0.40 (0.22–0.74)		0.60 (0.44–0.83)	
*Odds ratios (95% CI)*						
Reduced adherence: adherence <80% for at least one drug	1.16 (0.99–1.35)		1.21 (1.02–1.44)		1.18 (1.05–1.32)	

Covariate-adjusted hazards of changes in drug discontinuation, switching, and covariate-adjusted odds of reduced drug adherence after reaching the coverage gap spending threshold among propensity score matched beneficiaries. Adjusted for the number of physician visits and hospitalizations, drugs used, drug spending, and Charlson comorbidity score in the postbaseline, predoughnut hole period after PS matching for baseline characteristics, which included: age, gender, race, region of the US, rural/urban residence, median household income, Charlson comorbidity score, number of office-based drug infusions, physician visits and hospitalizations, Medicare Parts A and B spending, and diagnosis of cancer, RA, cardiovascular conditions (atrial or ventricular fibrillation, hypertension, hyperlipidemia, hypercholesterolemia, myocardial infarction, angina, atherosclerosis, or congestive heart failure), depression, dementia, and/or diabetes.

a *n* beneficiaries with at least one drug available at cohort entry.

## Discussion

In this paper we have shown that one-third of Medicare beneficiaries reached the coverage gap spending threshold in an average of 7 mo after enrollment. Beneficiaries who received no financial assistance to help pay drug costs after reaching the threshold were two times more likely to discontinue a drug but were 40% less likely to switch a drug compared to beneficiaries who did receive financial assistance. After accounting for a beneficiary's complete drug regimen, beneficiaries who received no financial assistance were 18% more likely to reduce their drug adherence. These surprising findings mean that when faced with the responsibility of paying 100% of their drug costs, beneficiaries discontinued therapy frequently or reduced adherence but were less likely to switch to less expensive or generic drugs. Among the cardiovascular drugs, there was a 2.6-fold increased likelihood of discontinuing a branded cardiovascular drug and a 1.8-fold increased likelihood of discontinuing a generic cardiovascular drug but no effect modification by brand/generic status. These results strongly suggest that increased discontinuation rates among the exposed were not driven by drug price alone.

Recent trends in drug insurance design have focused on making consumers more sensitive to drug costs. Our results demonstrate that while a blunt cost-sharing mechanism like the coverage gap does raise consumer sensitivity, it produces surprising consequences. Instead of incentivizing beneficiaries to switch to lower-priced or generic drugs, entry into the coverage gap resulted in an abrupt discontinuation of or reduced adherence to drugs among elderly Medicare beneficiaries. These results echo those of other studies that demonstrated that blunt measures had adverse effects on drug utilization and adherence [6],[16],[17] and are also in line with findings from Medicare Advantage Part D studies that observed increased rates of drug discontinuation [19],[20],[22] and adherence [19]–[21] but did not observe higher rates of drug switching to generics [22] during the coverage gap. A growing body of literature from diverse settings describes the adverse clinical consequences of stopping or reducing adherence to drugs in response to drug benefit caps, gaps in coverage, and high deductibles. [6],[17],[35],[36]. For example, abrupt increases in drug cost-sharing in Quebec, Canada resulted in a 9% decrease in essential drug use and a 7% increase in serious adverse events.[6]. A three-drug per month reimbursement limit on elderly Medicaid patients in New Hampshire resulted in a doubling of nursing home admission rates compared to a comparator US state [36]. In Germany, physicians who were required to pay for drug costs that exceeded a fixed budget discontinued their patients' medications more frequently, and these discontinuations may have led to increased hospitalization rates [37],[38]. Taiwan observed 2%–10% decreases in prescription costs and prescriptions when it introduced flat reimbursement rates to prescribing physicians [1].

An alternative strategy that may help beneficiaries forestall entry into the coverage gap is the initial prescription of generic or preferred medications, which has been associated with lower costs and better adherence over time [2],[39]. In British Columbia, introduction of a reference-drug program for angiotensin-converting enzyme (ACE) inhibitors was associated with a 24% decrease in drug discontinuation, no changes in health status or health systems use, and government savings of $6.7 million during the first year [2],[40],[41]. Value-based insurance design (VBID), in which patients' cost-sharing is reduced for medications that provide high benefits relative to costs, is a second potential strategy. Recent US studies observed 3%–4% increases in adherence when copayments for chronic medications were substantially reduced or eliminated [42],[43].

Our study has several strengths that enhance the validity of findings. Unlike previous studies [13],[20],[21],[22], we used multivariate PS-matched cohorts and additional adjustment for drug use and drug spending just prior to the coverage gap spending threshold. These measures strengthen our ability to compare beneficiaries who did and did not experience a gap in coverage. While unmeasured confounding may remain because of the limitations of the PS technique, its combined effect would need to be very strong to explain away the magnitude of the effect we observed [44]. For example, to explain even the 64% increased risk of drug discontinuation, the lower bound of the 95% CI, the odds of association between the unmeasured confounder and exposure would have to be at least an unrealistic 38.1, assuming a prevalence of the unmeasured confounder of 20% and a relative risk association of two between the unmeasured confounder and drug discontinuation (see Text S1 for calculations). Ours is also the first study to use linked prescription and health care claims from beneficiaries enrolled in heterogeneous stand-alone Part D plans and as such, our findings are generalizable to the 70% of Part D beneficiaries enrolled in such plans [23].

To assess whether any potential interdependence between the discontinuation and switching outcomes was indeed responsible for the opposite results of increased discontinuation but decreased switching among the exposed compared to the unexposed, we conducted a competing risks analysis. The competing risks analysis confirmed our findings. Based on each outcome's defined period of follow-up, this is not surprising. Study follow-up for the discontinuation outcome began 30 d after reaching the coverage gap spending threshold in order to allow for each drug's days supply to run out, thus avoiding immortal person-time bias [45]. In contrast, study follow-up for the switching outcome began 1 d after reaching the coverage gap spending threshold, as a drug could be switched before the days supply ran out, so there were 29 additional d during which there was no competing risk of discontinuation. Therefore, switching was not preempted by discontinuation but rather appears to be undertaken independently. It may be that beneficiaries who are aware that they will be exposed if they reaching the coverage gap spending threshold begin to switch their medications to lower-cost or generic versions before rather than after reaching the threshold in order to prevent or forestall coverage gap entry. However, surveys in 2006 and 2007 revealed that even when Part D beneficiaries were aware of the coverage gap, they frequently indicated that they did not understand how it worked or how to know whether they were at risk of entering the gap, reducing the likelihood of this early switching [46]–[48]. Again, these data suggest that when faced with a blunt cost sharing mechanism like the coverage gap, exposed beneficiaries were not able to navigate reducing their drug spending through switching drugs but instead simply stopped taking them.

In examining drug discontinuations and switches, other modeling approaches, such as a multistate model, in which beneficiaries could switch among the outcomes over time, are possible [49]. However, we focused on the first drug utilization change after a person enters the coverage gap because this first change is most closely temporally associated and further changes over time are less likely to be related. Because of the sparseness of outcomes, we were unable to calculate HRs for drug discontinuation and switching for several drug classes. Finally, during the first 3 mo of 2006, many Part D plans relaxed their coverage and cost-sharing requirements to ease beneficiaries into the new program. This was not the case in 2007. Therefore, in our pooled analyses, we may obscure differences among beneficiaries who enrolled in Part D during 2006 versus 2007, although a test of heterogeneity by year did not indicate a difference between years.

The adverse clinical consequences of stopping or reducing adherence to essential medications can be both severe and costly. Our results indicate that beneficiaries faced with increased out-of-pocket cost burdens during the Part D coverage gap are twice as likely to discontinue and more likely to reduce adherence to their medications but not to switch medications. At the population level, an estimated 18,000 additional patients discontinued ≥one medication because of an absence of financial assistance in the coverage gap period. Given the potential adverse health consequences of such discontinuations, changes to the coverage gap's structure are needed. The 2010 US Patient Protection and Affordable Care Act's Part D provisions will eliminate the coverage gap period incrementally by 2020, but beneficiaries may still be at risk of decreased drug utilization and adverse clinical consequences during that time. In contrast to blunt cost-sharing approaches such as the coverage gap feature, more nuanced, clinically informed insurance strategies that specifically promote the use of drugs with high benefit and low cost may hold the most promise for governments and insurers seeking to improve the health of their citizens while reigning in drug costs.

## Supporting Information

Text S1 **Supplementary information.** (1) Beneficiary group assignment algorithm; (2) diagnosis codes, definitions, and drugs and drug classes considered in our study; (3) drugs considered to have the same indication; (4) PS matched results; (5) sensitivity analyses for unmeasured confounding.
(DOC)

## Abbreviations

CI: confidence interval
PDC: proportion of days covered
PS: propensity score
RA: rheumatoid arthritis
