# Correction: Changes in Drug Utilization during a Gap in Insurance Coverage: An Examination of the Medicare Part D Coverage Gap

**DOI:** 10.1371/journal.pmed.1001716

**Published:** 2014-08-13

**Authors:** The PLOS Medicine Staff

Since the manuscript’s publication, the authors have been working on other, unrelated studies using the same database. The authors found results in these new, unrelated studies to be implausible and so undertook an intensive, several weeks-long investigation to determine whether the common database was in some way incorrectly assembled or was missing data. They found that a programmer in the group had failed to load 8 files of prescription and claims data into the dataset. This mistake occurred despite quality checks and resulted in the under-reporting of prescription drug use in the database.

The authors have now re-run all analyses for this study. In particular, because cohort entry in the Part D coverage gap study depends on prescription drug use, they now identify a larger number of patients and a larger number of drugs that can be studied. As a result, these analyses differ from those originally presented. The manuscript’s main message remains the same.

The authors sincerely apologize for these data issues and provide a corrected version of the article (Manuscript S1), Figure 2, and Text S1 below.

**Figure 2:**
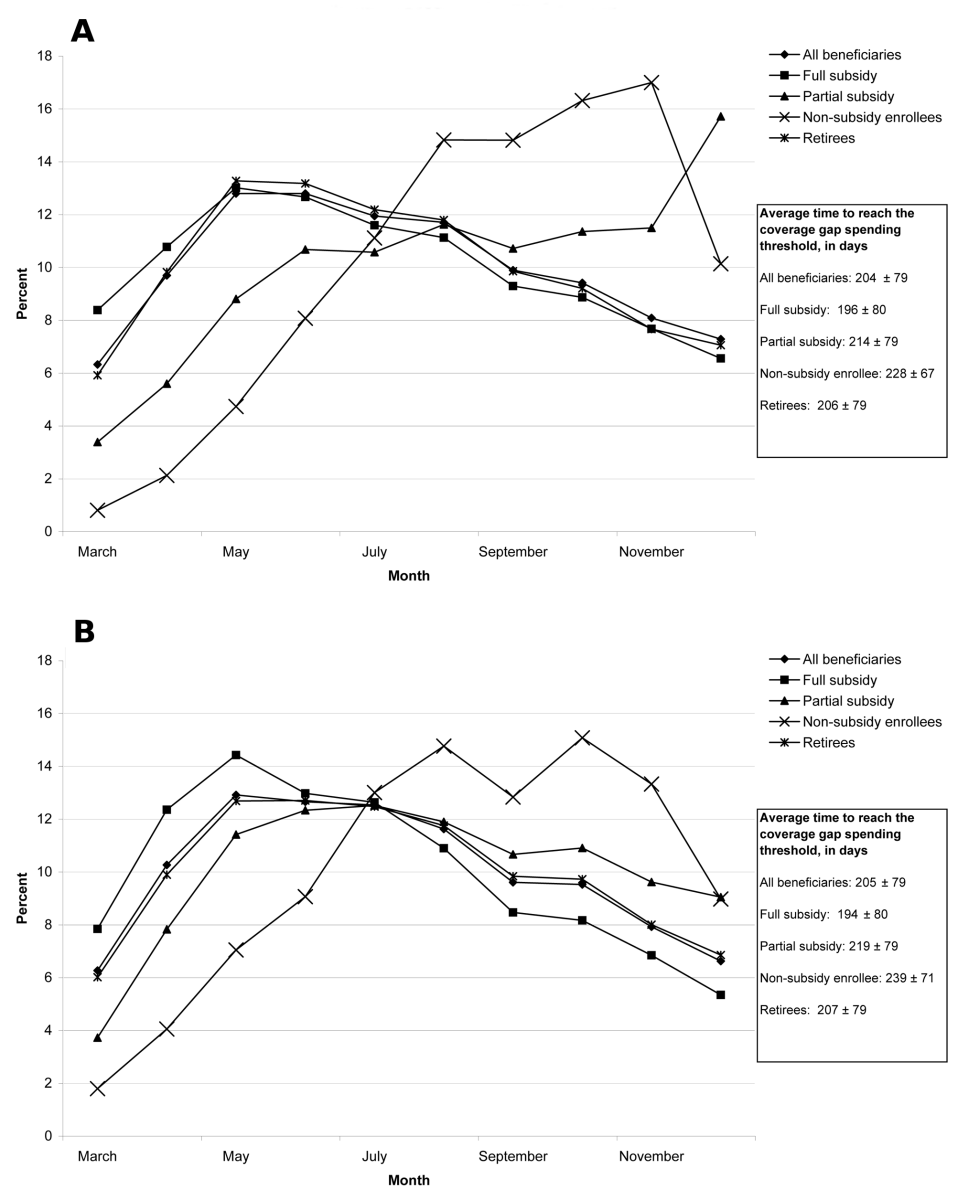
(A) Proportion of beneficiaries reaching the coverage gap spending threshold in each month in 2006, by beneficiary group. (B) Proportion of beneficiaries who reach the coverage gap spending threshold in each month in 2007, by beneficiary group**.**

## Supporting Information

Text S1. Supplementary Information Includes (1) beneficiary group assignment algorithm; (2) diagnosis codes, definitions, and drugs and drug classes considered in our study; (3) drugs considered to have the same indication; (4) PS matched results; (5) sensitivity analyses for unmeasured confounding.
(DOC)

Manuscript S1. Corrected Manuscript Text Original text with corrections to tables included.
(PDF)
