# Supplementary material for: Changes in Drug Utilization during a Gap in Insurance Coverage: An Examination of the Medicare Part D Coverage Gap
Source: PLoS Med. 2011 Aug 16;8(8):e1001075. doi: 10.1371/journal.pmed.1001075 (PMC3156689; doi:10.1371/journal.pmed.1001075)
Supplement: Text S1 — Supplementary information. (1) Beneficiary group assignment algorithm; (2) diagnosis codes, definitions, and drugs and drug classes considered in our study; (3) drugs considered to have the same indication; (4) PS matched results; (5) sensitivity analyses for unmeasured confounding. (DOC) [file pmed.1001075.s001.doc]

**Table 1. Beneficiary group assignment**

|  | **Deductible** | **Initial coverage period** | **Coverage gap period** | **Catastrophic coverage period** | **Exposure status in the coverage gap spending threshold analysis** |
| --- | --- | --- | --- | --- | --- |
| **Full subsidy beneficiaries**  [Income < $7,500 (single) or $12,000 (married) in 2006; income < $7,620 (single) or $12,190 (married) in 2007] | $0 | Co-insurance < $5 in 2006, < $5.35 in 2007 | Co-insurance < $5 in 2006, < $5.35 in 2007 | $0 | Unexposed: receive financial assistance to pay for drugs during the coverage gap |
| **Partial subsidy beneficiaries**  [Income < $11,500 (single) or $23,000 (married) in 2006; income < $11,710 (single) or $23,410 (married) in 2007] | Plan-dependent; $50 in 2006; $53 in 2007 in defined standard plan. | Plan-dependent; Co-insurance < 15% | Plan-dependent; Co-insurance < 15% | < $2 generic/$5 brand in 2006; < $2.15 generic/$5.35 brand in 2007 | Unexposed: receive financial assistance to pay for drugs during the coverage gap |
| **Non-subsidy enrollees**  [Do not receive any subsidy] | Part D plan-dependent; defined standard was $250 in 2006; $265 in 2007 | Part D plan-dependent; defined standard was 25% co-insurance in both 2006 and 2007 | 100% of drug costs (unless plan was enhanced alternative) | The greater of 5% or $2 generic/$5 brand in 2006; the greater of 5% or $2.15 generic/$5.35 brand | Exposed: do not receive any financial assistance during the coverage gap |
| **Retirees** [not enrolled in a Part D plan] | Cost-sharing throughout the benefit is plan-dependent. Because we have no identifier for retiree plan nor information about the cost-sharing structure, we cannot categorize these plans | | | | Unexposed: receive financial assistance to pay for drugs during the coverage gap |

Benefit type information drawn from:

1. Disabled and elderly health programs group, Centers for Medicare and Medicaid Services. Memorandum: 2007 resource standards. [www.nasmd.org/SMD_letters/docs/SMD-letter-on-Part-D-low-income-subsidy-December-18.pdf](http://www.nasmd.org/SMD_letters/docs/SMD-letter-on-Part-D-low-income-subsidy-December-18.pdf). Accessed November 6, 2009.
2. Office of the Actuary, Centers for Medicare and Medicaid Services. Medicare Part D benefit parameters for standard benefit: annual adjustments for 2007. [www.cms.hhs.gov/MedicareAdvtgSpecRateStats/Downloads/2007_PartD_Parameter_Update.pdf](http://www.cms.hhs.gov/MedicareAdvtgSpecRateStats/Downloads/2007_PartD_Parameter_Update.pdf). Accessed November 6, 2009.

**Diagnostic definitions for 5 diseases of interest**

Rheumatoid arthritis (RA)

At least one diagnosis of rheumatoid arthritis [inpatient or outpatient] in the baseline year

- ICD-9 codes and validation: at least 1 inpatient or outpatient diagnosis of 714, 714.0
- Validation study: Losina E, Barrett J, Baron JA, Katz JN. Accuracy of Medicare claims data for rheumatologic diagnoses in total hip replacement recipients. *Journal of Clinical Epidemiology.* 2003;56(6):515-519.

Diabetes

At least one diagnosis of diabetes [inpatient or outpatient] in the baseline year

- ICD-9 codes: 250.0-250.9
- Validation study: Wilchesky M, Tamblyn RM, Huang A. Validation of diagnostic codes within medical services claims. *Journal of Clinical Epidemiology.* 2004;57(2):131-141.

Cardiovascular conditions

At least one diagnosis of a cardiovascular condition in the baseline year

- - Atrial fibrillation: ICD-9 code 427.31 as an inpatient diagnosis in the primary or secondary diagnosis field.

Validation study: Kokotailo RA, Hill MD. Coding of stroke and stroke risk factors using international classification of diseases, revisions 9 and 10. *Stroke.* 2005;36(8):1776-1781.

- - Hypertension: ICD-9 code 401.0 – 401.9 [inpatient or outpatient]

Validation study: Wilchesky M, Tamblyn RM, Huang A. Validation of diagnostic codes within medical services claims. *Journal of Clinical Epidemiology.* 2004;57(2):131-141.

- - Hyperlipidemia/hypercholesterolemia: ICD-9 272.0, 272.2, [inpatient or outpatient]
  - Cardiovascular disease: ICD-9 code 410.xx, 427.4, 427.5, 411.xx, 412.xx, 413.xx, 414.xx, [inpatient or outpatient]

Validation study: Newton KM, Wagner EH, Ramsey SD, et al. The use of automated data to identify complications and comorbidities of diabetes: a validation study. *Journal of Clinical Epidemiology.* 1999;52(3):199-207.

- - Congestive heart failure: ICD-9 428.0 – 428.9, 429.3 [inpatient or outpatient]

Validation study: Wilchesky M, Tamblyn RM, Huang A. Validation of diagnostic codes within medical services claims. *Journal of Clinical Epidemiology.* 2004;57(2):131-141.

Depression

At least one diagnosis of depression in the baseline year

- - ICD-9 codes: 296.2x, 296.3x, 309.0x, 309.1x, 311.xx [outpatient]
- Validation study: West SL, Richter A, Melfi CA, McNutt M, Nennstiel ME, Mauskopf JA. Assessing the Saskatchewan database for outcomes research studies of depression and its treatment. *Journal of Clinical Epidemiology.* 2000;53(8):823-831.

Dementia/Alzheimer’s disease

At least one diagnosis of dementia or Alzheimer’s disease in the baseline year

- ICD-9 codes: 290.x, 331.xx-331.2x [inpatient or outpatient]

**Table 2. Drugs and drug classes evaluated for each of the diseases**

| **Condition** | **Drug classes/individual drugs** |
| --- | --- |
| Diabetes | - Metformin - Sulfonylureas - Glitazones - Acarbose - Miglitol - Repaglinide - Nateglinide - Sitagliptin - Exenatide - Insulin |
| Depression | - Antidepressants |
| Dementia/Alzheimer’s disease | - Antipsychotics - Cholinesterase inhibitors - Memantine - Tacrine |
| Cardiovascular disease | - Warfarin - Statins - Niacin and fibrates - Bile acid sequestrants - ACE Inhibitors - Loop diuretics - Angiotensin receptor blockers - Aldosterone antagonists - Beta-blockers - Digoxin - Anti-platelet drugs - Thiazide diuretics - Calcium channel blockers - Potassium-sparing agents - Alpha blockers - Diazoxide - Metyrosine - Reserpine/mannitol hexanitrate - Acetazolamide |
| Rheumatoid arthritis | - Disease-modifying anti-rheumatic drugs (DMARDS) - Methotrexate - Gold salts - Penicillamine - Hydroxychloroquine - Sulfasalazine - Cyclosporine - Cyclophosphamide - Leflunomide |

**Drug switches**

**The following drug switches satisfy the criterion “Begins new drug in a different class but with the same indication as drug X.”**

**For patients with diabetes**

The following classes are interchangeable:

- ACE inhibitors and angiotensin receptor blockers

**For patients with cardiovascular disease**

The following classes/drugs are interchangeable:

- ACE inhibitors and angiotensin receptor blockers
- aldosterone antagonists and potassium-sparing agents

**For patients with Alzheimer’s disease/dementia**

The following classes are interchangeable:

- Cholinesterase inhibitors and memantine
- Cholinesterase inhibitors and tacrine
- Memantine and tacrine

**Table 3**. Multivariate propensity score model results: Odds of receiving no financial assistance to help pay for drug costs versus receiving financial assistance among beneficiaries who reached the coverage gap spending threshold in 2006 (Early Part D cohort) and in 2007 (Established Part D cohort)

|  | **Early Part D cohort**  N = 121,760  C statistic: 0.665 | **Established Part D cohort**  N = 95,731  C statistic: 0.670 |
| --- | --- | --- |
|  | *OR (95% CI)* | |
| Female gender | 1.21 (1.07 – 1.38) | 1.36 (1.18 – 1.57) |
| Age as of January 1 of study year |  |  |
| 65 – 74 | Reference | Reference |
| 75 – 84 | 1.03 (0.90 – 1.18) | 1.21 (1.04 – 1.40) |
| 85+ | 1.42 (1.19 – 1.69) | 1.71 (1.42 – 2.07) |
| Race |  |  |
| White | Reference | Reference |
| Black | 0.44 (0.30 – 0.64) | 0.41 (0.26 – 0.64) |
| Other | 0.34 (0.19 – 0.58) | 0.38 (0.21 – 0.69) |
| Urban residence | 1.00 (0.85 – 1.19) | 0.76 (0.64 – 0.91) |
| Region of the U.S. |  |  |
| West | Reference | Reference |
| Northeast | 2.51 (2.00 – 3.15) | 1.78 (1.41 – 2.24) |
| Midwest | 0.82 (0.64 – 1.06) | 0.50 (0.38 – 0.65) |
| South | 0.83 (0.65 – 1.05) | 0.89 (0.70 – 1.13) |
|  |  |  |
| Median household income, per $10,000 | 1.10 (1.07 – 1.14) | 1.11 (1.07 – 1.14) |
| Total Medicare Parts A, B spending, per $10,000 | 1.03 (0.99 – 1.07) | 1.05 (1.00 – 1.10) |
|  |  |  |
| Charlson comorbidity score | 0.99 (0.94 – 1.03) | 0.98 (0.94 – 1.03) |
| Number of physician visits | 1.01 (1.00 – 1.02) | 1.01 (1.00 – 1.02) |
| Number of hospitalizations | 0.91 (0.82 – 1.02) | 0.98 (0.87 – 1.10) |
| Number of office-based drug infusions | 1.01 (0.96 – 1.05) | 0.98 (0.92 – 1.05) |
|  |  |  |
| Diagnosis of cancer | 1.15 (0.97 – 1.37) | 1.07 (0.89 – 1.30) |
| Diagnosis of rheumatoid arthritis | 1.02 (0.74 – 1.39) | 1.09 (0.79 – 1.51) |
| Diagnosis of cardiovascular condition | 1.31 (1.02 – 1.67) | 1.14 (0.88 – 1.47) |
| Diagnosis of depression | 0.76 (0.63 – 0.93) | 0.71 (0.57 – 0.89) |
| Diagnosis of diabetes | 1.04 (0.91 – 1.19) | 0.95 (0.82 – 1.10) |
| Diagnosis of dementia | 0.79 (0.64 – 0.98) | 0.83 (0.66 – 1.04) |

**Sensitivity analysis**

**How strong does an unmeasured confounder have to be to fully explain the observed findings for the association between exposure to 100% of drug costs after reaching the Part D coverage gap spending threshold and drug discontinuation?**

(Following the logic and explanation of Schneeweiss, 2006.^25^)

The relationship between OR_EC_ and RR_CD_ for a given ARR, RR_CD_, P_C_, P_E_.

Where OR_EC_ = the odds ratio between the exposure and the confounder

RR_CD_ = the risk ratio between the confounder and the outcome

ARR = the apparent risk ratio observed between the exposure and outcome

P_C_ = the prevalence of the confounder in the population

P_E_ = the prevalence of the exposure

Walker^26^ showed that with a dichotomous confounder and a dichotomous outcome, the association between the confounder and the exposure can be measured using an odds ratio, which is a function of the prevalence of the confounder among the exposed (P_C1_) and the marginal prevalence of exposure (P_E_ ) and prevalence of the confounder (P_C_).

$$OR_{EC}=\frac{P_{C1}[1-P_{C}-P_{E}+P_{C1}]}{[P_{C}-P_{C1}][P_{E}-P_{C1}]}$$

Assuming no underlying association between exposure and death, the ARR is a function of P_C1_, P_E_ and P_C_, and the confounder-outcome association RR_CD_

$$ARR=\frac{P_{C1}[RR_{CD}-1]+P_{E}}{[P_{C}-P_{C1}][RR_{CD}-1]-P_{E}+1}*\frac{1-P_{E}}{P_{E}}$$

Using the ARR equation, we solve for P_C1_ and substitute the derived term for P_C1_ from the OR_EC_ equation in order to explore the relationship between OR_EC_ and RR_CD_.

We present two scenarios, one with a confounder prevalence of 20%, one with a confounder prevalence of 30%.

**Example 1.**

- Here, we test the strength of unmeasured confounding for both the point estimate, 2.00 and for the lower bound of the 95% confidence interval, 1.64.
- We assume a prevalence of the exposure of 1 in 6 and a prevalence of the confounder of 20%.
- The areas to the upper right of each curve indicate all values of RR_CD_ and OR_EC_ that would lead to an observed RR_ED_ of 1 (no association between exposure and drug discontinuation). For example, to explain away the 64% increased drug discontinuation with an RR_CD_ of 2, the strength of the OR_EC_ would need to be at least 38.12.

**Example 2.**

- Here, we test the strength of unmeasured confounding for both the point estimate, 2.00 and for the lower bound of the 95% confidence interval, 1.64.
- We assume a prevalence of the exposure of 1 in 6 and a prevalence of the confounder of 30%.
- The areas to the upper right of each curve indicate all values of RR_CD_ and OR_EC_ that would lead to an observed RR_ED_ of 1 (no association between exposure and death). For example, to explain away the 64% increased drug discontinuation with an RR_CD_ of 3, the strength of the OR_EC_ would need to be at least 7.60.
